# Inflammatory cytokines as predictors of therapeutic response to valproate in patients with comorbid alcohol use disorder and bipolar disorder

**DOI:** 10.3389/fpsyt.2026.1811475

**Published:** 2026-09-24

**Authors:** Ilya O. Blokhin, Dana P. Ascherman, Feng Miao, Lisa Harlow, Eleonore Beurel, Jin Cheng, Claes Wahlestedt, Antoine B. Douaihy, Ihsan M. Salloum

**Affiliations:** 1 Department of Psychiatry, Harvard University, Boston, MA, United States; 2 Department of Medicine, University of Pittsburgh, Pittsburgh, PA, United States; 3 Department of Psychiatry and Behavioral Sciences, University of Miami Miller School of Medicine, Miami, FL, United States; 4 Department of Medicine, University of Miami Miller School of Medicine, Miami, FL, United States; 5 Pittsburgh Veteran Affairs Medical Center, Pittsburgh, PA, United States; 6 Department of Psychiatry, University of Pittsburgh, Pittsburgh, PA, United States; 7 Institute of Neuroscience, University of Texas Rio Grande Valley School of Medicine, Harlingen, TX, United States

**Keywords:** alcohol use disorder, bipolar disorder, inflammation, valproate, extracellular particles

## Abstract

**Objective:**

Alcohol use disorder (AUD) is a chronic condition commonly associated with bipolar disorder (BD). We previously reported that valproate (VPA) can decrease drinking in some patients with comorbid AUD/BD. Predictors of treatment response to VPA, however, are yet to be elucidated, and it is not possible to prospectively identify patients who may benefit from the course of VPA. Both pathogenesis of AUD/BD and mechanism of action of VPA are related to inflammation (AUD/BD confers a pro-inflammatory state, while VPA has anti-inflammatory properties) which is associated with an altered count of plasma extracellular particles.

**Approach:**

Patients with co-occurring AUD/BD were recruited and treated with VPA for 3 months at an average dose of 1,000 mg a day. Clinical response was defined as a decrease in proportion of weekly heavy drinking days (defined as ≥5 drinks per day for men and ≥4 drinks per day for women). Levels of inflammatory cytokines in plasma were measured using MAGPIX Luminex. Counts of extracellular plasma particles were determined using flow cytometry with size beads.

**Results:**

We found that plasma levels of C-reactive protein (CRP) at baseline were significantly increased in patients who responded to VPA. We did not find the difference in counts of extracellular particles between responders and non-responders. In line, we observed that treatment with VPA conferred an anti-inflammatory effect but did not alter counts of plasma particles.

**Conclusion:**

This pilot study indicates that patients with AUD/BD who have increased levels of CRP are more likely to benefit from the treatment with VPA.

## Introduction

Alcohol use disorder (AUD) is a chronic condition characterized by a problematic pattern of alcohol use leading to clinically significant impairment. Alcohol consumption accounts for 2.6 million deaths per year or 4.7% of all deaths worldwide, according to the report from the World Health Organization (1). In the United States, 14% of adults currently meet criteria of AUD, and 29% met AUD criteria once during their lifetime (2–4). Annual cost of AUD and alcohol-related disorders has reached ~$250 billion (5), and prevalence of AUD continues to rise (6, 7). Despite considerable progress has been made in achieving abstinence (8), ~70-80% of patients still relapse within a year following treatment (9, 10).

Bipolar disorder (BD) has the highest prevalence of co-occurring with AUD and other substance use disorders, compared to other mental illnesses (11–13). Data from several studies indicated that the prevalence of AUD in patients with BD ranges from 30% to 50%, depending on population (14, 15). Co-occurring AUD/BD is also associated with severe morbidity and increased suicide risk (16). Effective therapeutic interventions to target AUD in AUD/BD population are lacking. We have previously observed that some patients with AUD/BD treated with valproic acid (VPA) had significantly lower proportion of heavy drinking days and fewer drinks per heavy drinking day compared to placebo (17). Likewise, Brady et al. (18) reported that VPA prevented relapse to heavy drinking in patients with AUD. Predictors of therapeutic response to VPA in AUD/BD remain unknown which makes it difficult to identify patients who would likely benefit from the treatment with VPA.

Recent evidence suggests that AUD and BD increase the production of pro-inflammatory cytokines while VPA reduces the levels of pro-inflammatory factors. For example, AUD is associated with an increased plasma levels of such inflammatory markers as tumor necrosis factor (TNF), CCL2, and CCL5 (19–21). There is also an association between BD and increased pro-inflammatory cytokines including C-reactive protein (CRP), IL-2, IL-6, and TNF (22–27). VPA, on the other hand, has been known to diminish inflammatory indices (28, 29). Inflammation is closely intertwined with circulating plasma vesicles which appear to promote inflammation. Pro-inflammatory state triggers an increased production of extracellular particles (30, 31), and shed particles constitute a rich source of pro-inflammatory compounds, thus further amplifying inflammation (32, 33).

We hypothesized that patients with AUD/BD who respond to VPA have higher baseline inflammatory indices and elevated counts of plasma particles and that VPA has an anti-inflammatory effect and decreases the counts of circulating particles.

## Materials and methods

### Subjects

Subjects with DSM-IV-defined diagnoses of AUD and BD (AUD/BD) were included in the study and represented a subpopulation derived from our previous investigations which evaluated the efficacy of valproic acid in patients with co-morbid bipolar disorder and AUD (17, 34–36). Briefly, patients were recruited from clinical programs as well as via community advertisements during 2007–2014 period. The study was conducted at the Addiction Medicine Services of the Western Psychiatric Institute at the University of Pittsburgh. Patients had to meet DSM-IV diagnostic criteria of both bipolar disorder and AUD (diagnoses were determined using the Mini-International Neuropsychiatric Interview (37), administered by a trained research staff) and had to be drinking alcohol at the moment of enrollment (patients in partial or full remission were not included). This pilot study included two parts. In the first part, we addressed if treatment with VPA affects plasma levels of cytokines and counts of plasma vesicles; in the second part, we aimed to identify if cytokines of interest can serve as predictors of clinical response to VPA. 9 subjects were included in the first part, and 11 subjects were included in the second part (5 non-responders and 6 responders). Blood samples included in this study were collected between May 2009 and February 2013. Study protocol was approved by the Institutional Review Boards of the University of Pittsburgh. Subjects were excluded if they had any other major psychiatric disorder, if they had any severe or unstable medical conditions, or if they were pregnant or lactating. Furthermore, since VPA can be hepatotoxic (38), subjects with the elevation of liver function enzymes (γ-glutamyl transpeptidase, aspartate aminotransferase, alanine aminotransferase, and alkaline phosphatase) greater than 3-fold above the reference range were excluded.

### Treatment with VPA

Patients received a course of VPA for 3 months at the average dose of 1,000 mg a day. The use of alternative mood stabilizers (lithium, carbamazepine, or lamotrigine) was not allowed. The use of naltrexone was allowed. Liver function tests (including γ-glutamyl transpeptidase (γ-GTP), aspartate aminotransferase (AST), alanine aminotransferase (ALT), and alkaline phosphatase (ALP)) and trough VPA plasma levels were evaluated periodically. Blood was collected from the median cubital vein prior to the initiation of treatment with VPA and throughout the study. Plasma samples were isolated by centrifugation (1,000 x g for 15–20 minutes) and stored at -80°C until use.

### Evaluation of clinical response

Alcohol use was assessed using the Timeline Follow-Back for Recent Drinking (39). The use of other drugs was monitored through regular urine drug screens. The primary alcohol use outcome used in our original studies was the change in proportion of weekly heavy drinking days (defined as ≥5 drinks per day for men and ≥4 drinks per day for women), in accordance with our previous studies (17, 40, 41). In the current work, response was defined as the complete abstinence from alcohol during last 4 weeks of treatment.

### Measurement of plasma cytokines

Plasma levels of cytokines, chemokines, and growth factors were measured on a MAGPIX Luminex using a commercially available multiplex assays such as ProcartaPlex Multiplex Immunoassay (Thermo Fisher Scientific), Milliplex Map Kit (Millipore), and Magnetic Luminex Assay (R&D Systems), in accordance with the manufacturers’ recommendations. Assays were checked for quality control to fit the standard curves. A standard curve was run for each lot, and samples were normalized to the averaged standard curve values using standard software from the Luminex.

### Isolation of extracellular particles

Plasma was thawed on ice, and extracellular particles were pelleted using centrifugation at 10,000 g for 30 minutes at 4°C. Nomenclature pertaining to the experiments on extracellular particles was in accord with the most recent update of Minimal Information for Studies of Extracellular Vesicles (42).

### Quantitative real-time PCR

RNA from extracellular particles was isolated by Qiagen RNeasy kit, in accordance with manufacturer’s instructions. DNase treatment was performed using RNase-Free DNase Set (Qiagen). RNA profile, quality, and concentrations were determined by Nanodrop 2000; samples with RNA Integrity Number (A260:A280 ratio) between 1.9 – 2.1 were included in the study. RNA was reversely transcribed using High-Capacity cDNA Reverse Transcription Kit (Thermo Fisher Scientific), and qRT-PCR was performed on QuantStudio 6 Flex (Thermo Fisher Scientific) using probe-based assays from Integrated DNA Technologies.

### Flow cytometry

Extracellular particles were counted in Flow Cytometry Shared Resource of the Sylvester Comprehensive Cancer Center at the University of Miami Miller School of Medicine. Flow Cytometry Size Calibration Kit (F13838, Thermo Fisher Scientific) was used to determine the size of particles. 123count eBeads Counting Beads (01-1234, Thermo Fisher Scientific) were used to determine the absolute counts of extracellular particles; counting beads were 7 μm in diameter and emitted a signal in the violet (405 nm) and blue (488 nm) lasers. Samples and 123count eBeads Counting Beads were mixed in 1:1 ratio (100 μl of each) and added to 0.3 ml of PBS. Sample acquisition was performed on a BD FACS Aria Fusion machine equipped with a violet (405 nm), blue (488 nm), yellow-green (561 nm), and red (640 nm) lasers. Forward Scatter and Side Scatter parameters were set on a logarithmic scale, and the Forward Scatter threshold was set at the lowest limit of 200. The Forward Scatter and Side Scatter voltages were then adjusted to set the Flow Cytometry Size Calibration beads on scale with the 10 μm diameter microspheres set at the highest scale for both Forward Scatter and Side Scatter. The voltages on the FITC and PerCP parameters where then adjusted to bring the 123count eBeads Counting Beads on scale.

### Statistical analysis

Statistical analysis was performed using two-tailed Student’s *t* test. Data are expressed as mean ± SEM unless otherwise stated. Differences with *P* < 0.05 were considered to be statistically significant.

## Results

### Effect of VPA on plasma levels of inflammatory markers

There is some evidence that VPA affects inflammatory pathways and decreases inflammation (43). Specifically, VPA alters such cytokines as IL-5, IL-1β, IL-6, IL-10, TNF, and CCL2 (44, 45). We therefore set out to determine if treatment with VPA may alter plasma levels of inflammatory markers in patients with AUD/BD. We found that a course of VPA treatment decreased levels of pro-inflammatory CRP and increased levels of the metalloprotease MMP-10 (P < 0.05, **Figure 1**). Most of pro- and anti-inflammatory cytokines, however, remained unaffected, although there was a trend to increased concentrations of IL-10 and CCL-2 (e.g. MCP-1) (P > 0.05, **Table 1**).

**Figure 1:**
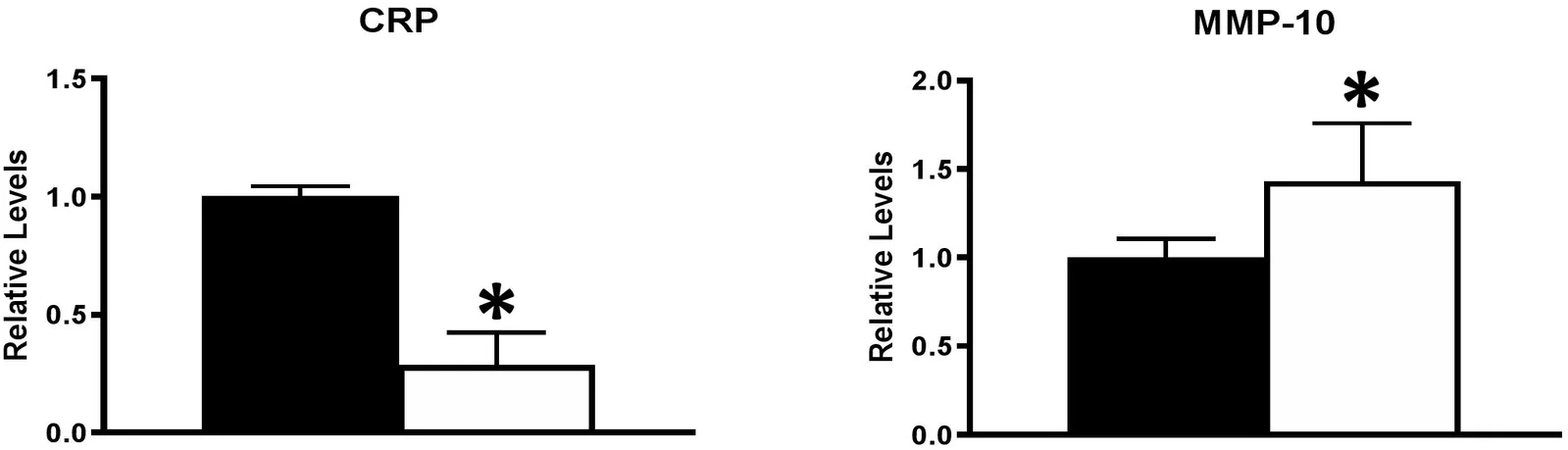
Plasma levels of C-reactive protein (CRP) and matrix metalloproteinase-10 (MMP-10) before and after treatment with valproate (black and white bars, respectively). P < 0.05.

**Table 1:** Relative change in plasma levels of cytokines after treatment with valproate.

Cytokine	Fold change	p value
CCL-3/MIP-1a	0.87	0.8
CXCL-12/SDF-1a	0.99	0.9
IL-27	1.00	0.9
CXCL-10/IP-10	1.12	0.7
IL-8	1.07	0.9
IL-10	1.95	0.1
CCL-11/Eotaxin	1.47	0.4
IL-17a	1.08	0.6
CCL-5/RANTES	0.43	0.4
CCL-4/MIP-1b	1.06	0.8
CCL-2MCP-1	1.38	0.1
IL-9	1.00	0.9
CXCl-1/GRO-a	1.31	0.7
IL-23	0.75	0.7
IL-21	1.06	0.9
IL-22	1.03	0.9
IL-1a	1.58	0.2
IL-2	1.15	0.2
IL-4	2.25	0.3
IL-6	3.04	0.3
TNF	1.27	0.2
C5/C5a	1.02	0.9
C2	0.86	0.4
C3a	0.85	0.4

### Inflammatory markers as predictors of therapeutic response to VPA in patients with AUD/BD

Currently, there are no predictors of treatment response in isolated AUD, with combined AUD/BD remaining even more challenging to approach with reliable prognosticators. Results from several studies indicate that inflammatory cytokines are frequently increased in patients with AUD (46), BD (22, 23, 47), and in patients with combined psychiatric illnesses such as AUD/major depressive disorder (48). We therefore screened panels of pro- and anti-inflammatory markers in patients with AUD/BD to address if plasma concentrations of cytokines prior to the treatment with VPA may predict a therapeutic response. Among recruited patients with AUD/BD, approximately 50% demonstrated decreased use of alcohol in response to VPA. Responders were defined as those individuals who were either abstinent or with significant decrease in weekly heavy drinking days (=>5 sd/d male; =>4 sd/d, female). We observed that responders had higher initial levels of pro-inflammatory cytokine CRP (P < 0.05, **Figure 2**) while other cytokines did not correlate with the clinical response to VPA (P > 0.05, **Table 2**).

**Figure 2:**
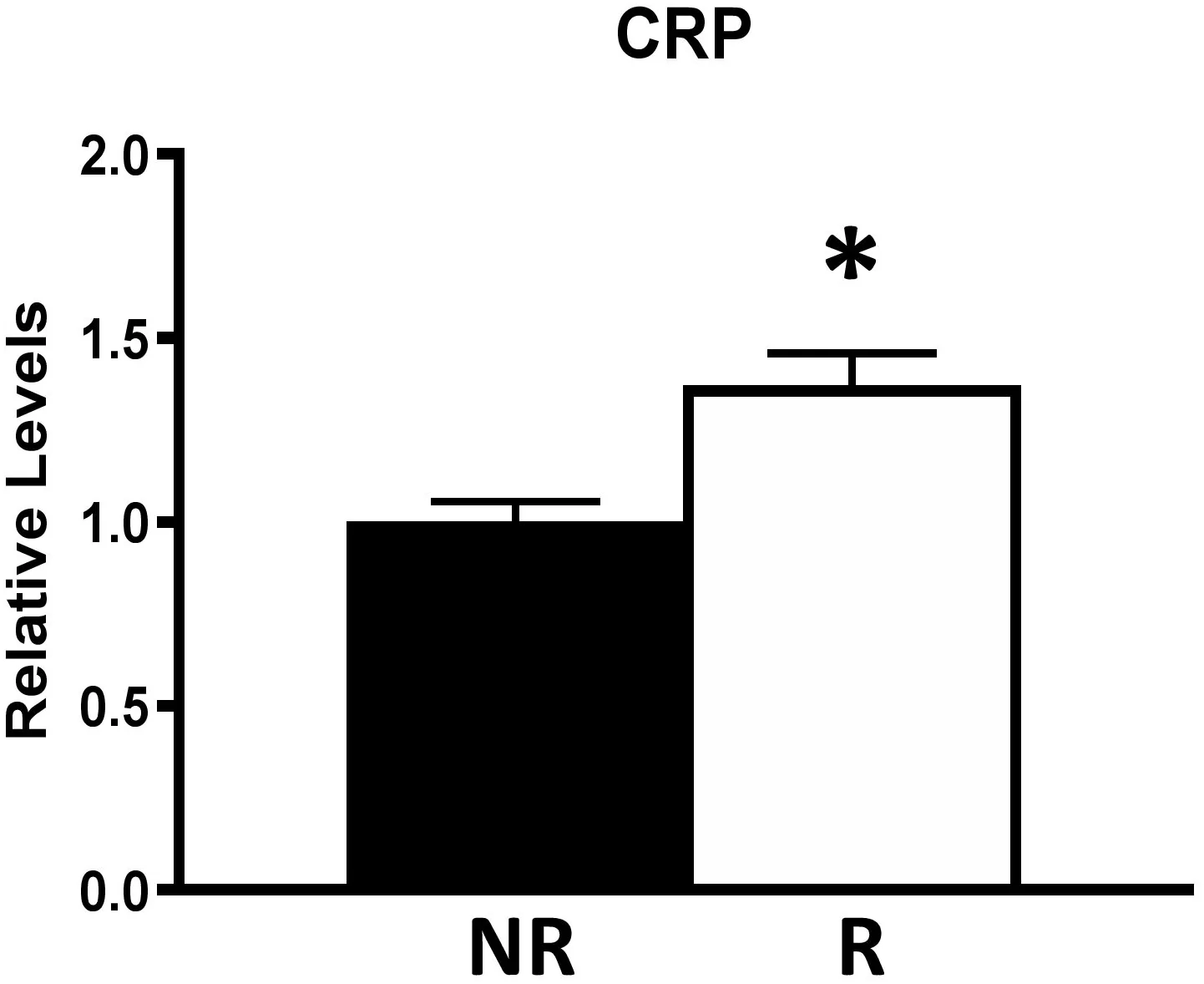
Plasma levels of C-reactive protein (CRP) before the initiation of treatment with valproate. NR, non-responders; R, responders. Values expressed as a relative to the group of non-responders. P < 0.05.

**Table 2:** Cytokines not predictive of clinical response to valproate.

Cytokine	Fold change	p value
Eotaxin	0.87	0.8
IL-17	0.53	0.5
IP-10	1.04	0.9
MCP-1	1.29	0.3
MIP-1b	1.34	0.4
RANTES	1.01	0.9
SDF-1a (13)	2.03	0.1
C2	0.96	0.4
C3	1.02	0.8
C5/C5a	0.62	0.2
MMP-10	0.82	0.5
IL-1a	0.93	0.9
IL-2	0.62	0.6
IL-4	1.07	0.8
IL-6	1.29	0.6
TNF	0.65	0.6

Values expressed as a relative of the group of responders to the group of non-responders.

### Effect of VPA on extracellular particles

The effect of VPA on counts of plasma particles is unknown. Given that VPA confers a pro-inflammatory effect (43) and that circulating particles can induce and amplify inflammation (32, 49, 50), we sought to ascertain whether VPA affects counts of extracellular particles in AUD/BD cohort. First, we isolated the particles from plasma using centrifugation and determined RNA distribution using Nanodrop 2000. In contrast to previous reports (51), RNA profile in extracellular particles was strikingly different from that in nucleated cells (**Figure 3**). To ensure RNA integrity, we measured expression of several genes by quantitative real-time PCR. While we observed that all measured genes were robustly expressed indicating high quality of RNA (**Figure 4A**), we unexpectedly found that expression of genes tended to be much less in patients with detectable plasma levels of VPA compared to those with undetectable VPA levels (collected from a small additional cohort of patients who were likely non-compliant with the treatment protocol, P = 0.1, **Figure 4B**). We reasoned that two scenarios could be responsible for such a trend. First, VPA could lower the levels of extracellular particles in plasma which would be in line with a previous report describing anti-apoptotic effect of VPA (28) and with the observed anti-inflammatory action of VPA (**Figure 1**). Second, VPA could decrease levels of RNA cargo in the particles. To differentiate between two scenarios, we counted extracellular particles in plasma using flow cytometry with size beads and found that their counts did not differ in patients prior to and after the treatment with VPA (P > 0.05, **Figure 5**).

**Figure 3:**
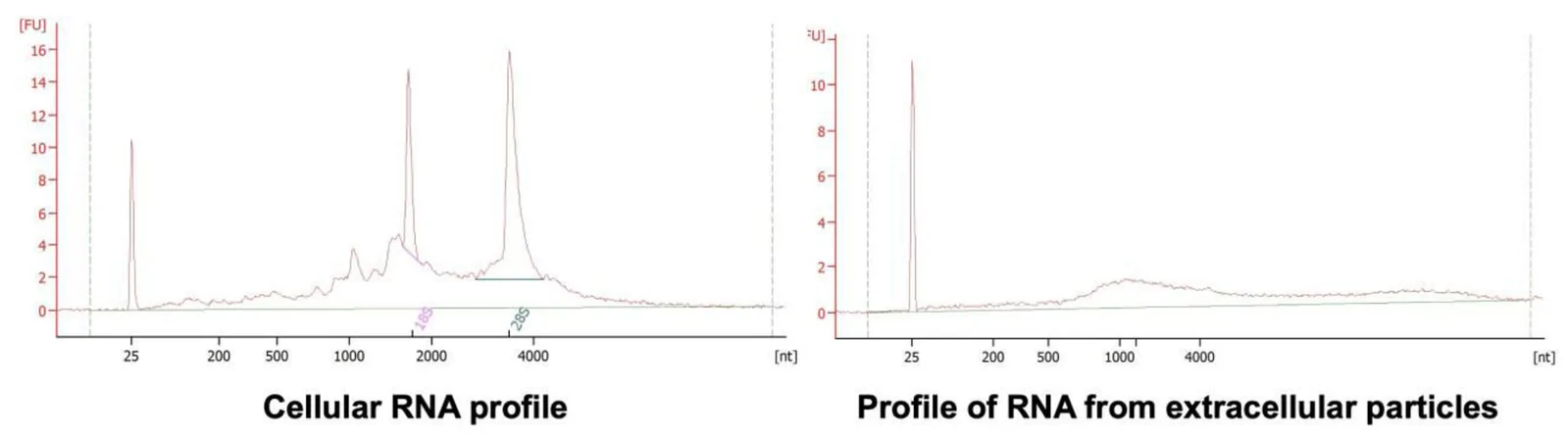
Profile of RNA from cells and extracellular particles.

**Figure 4:**
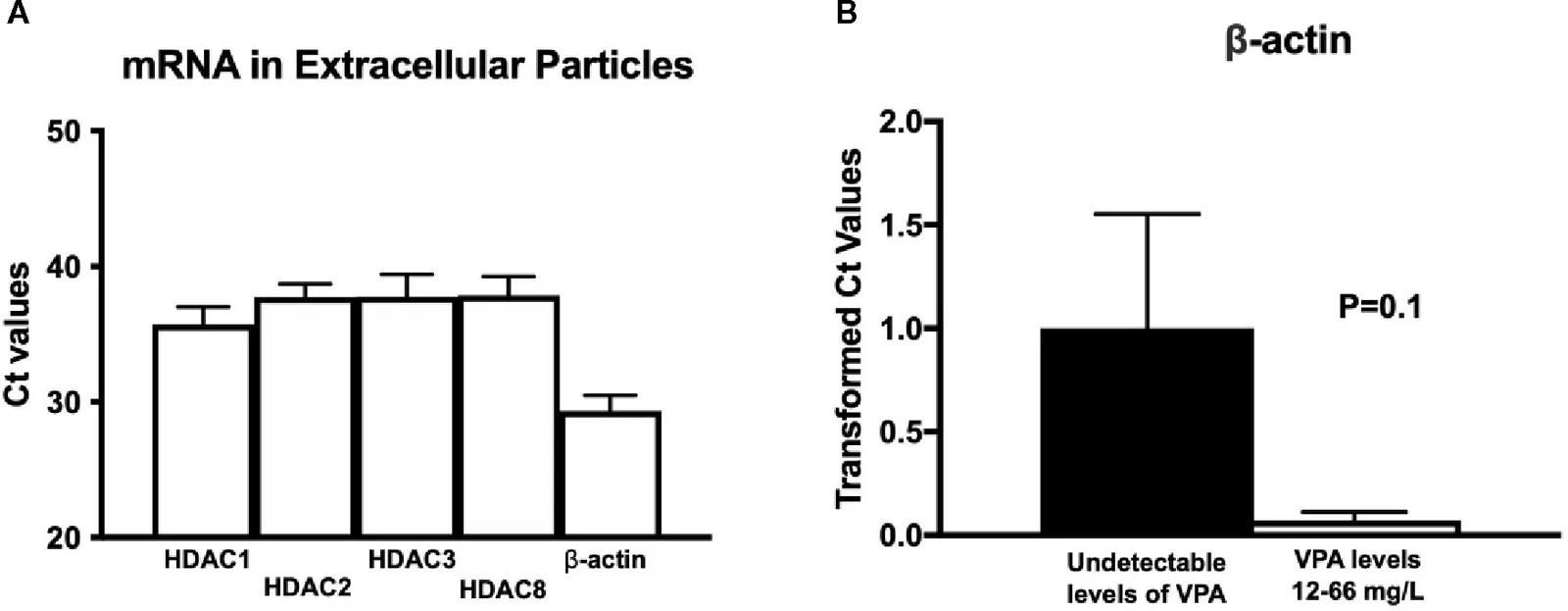
Expression of histone deacetylases (HDACs) and B-actin in extracellular particles **(A)**. Levels of B-actin in extracellular particles in patients with detectable and undetectable levels of valproic acid (VPA) **(B)**.

**Figure 5:**
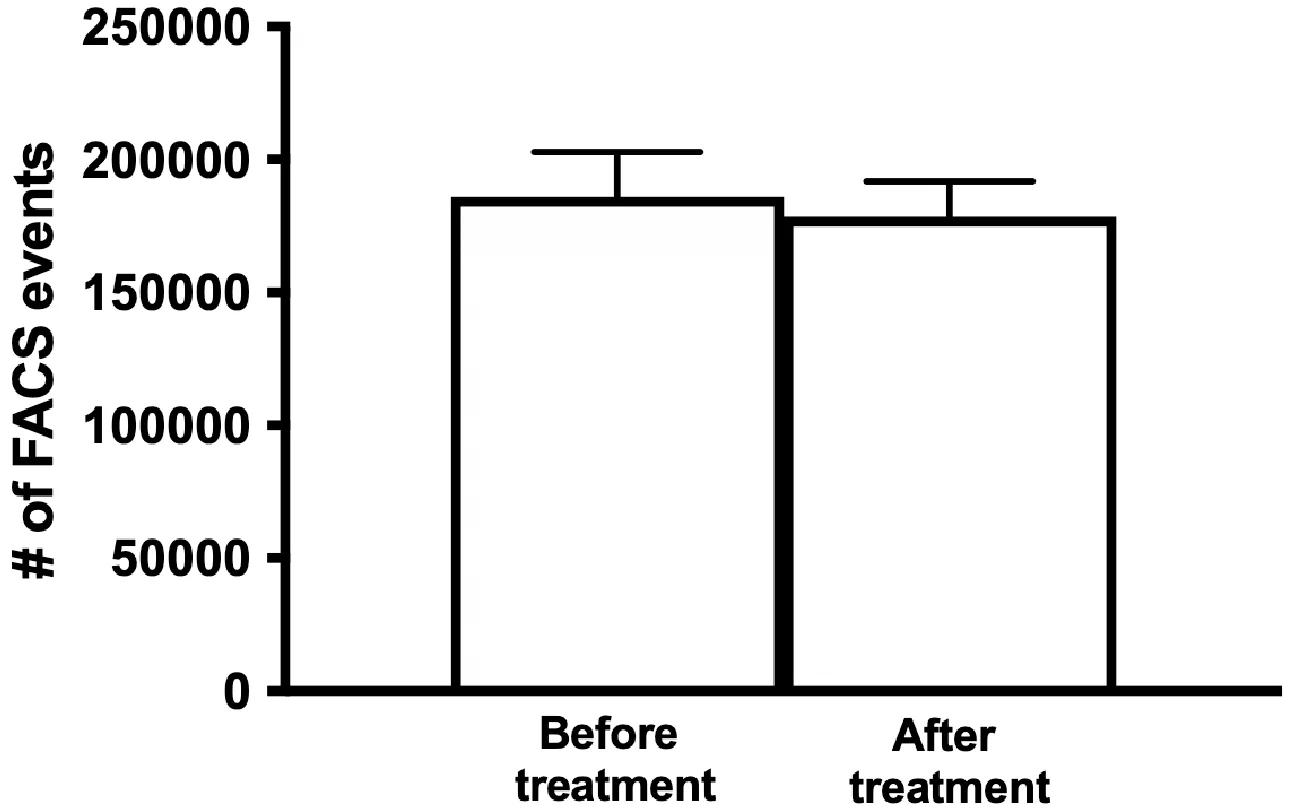
Plasma counts of extracellular plasma particles before and after treatment with valproate (per μl).

### Extracellular particles as predictors of therapeutic response to VPA in patients with AUD/BD

Next, we aimed to address if plasma levels of particles can predict therapeutic response to VPA in patients with AUD/BD. No difference in their counts was detected between responders and non-responders to VPA (**Figure 6**) indicating that extracellular particles in plasma are unlikely to serve as prognosticators of therapeutic response to VPA.

**Figure 6:**
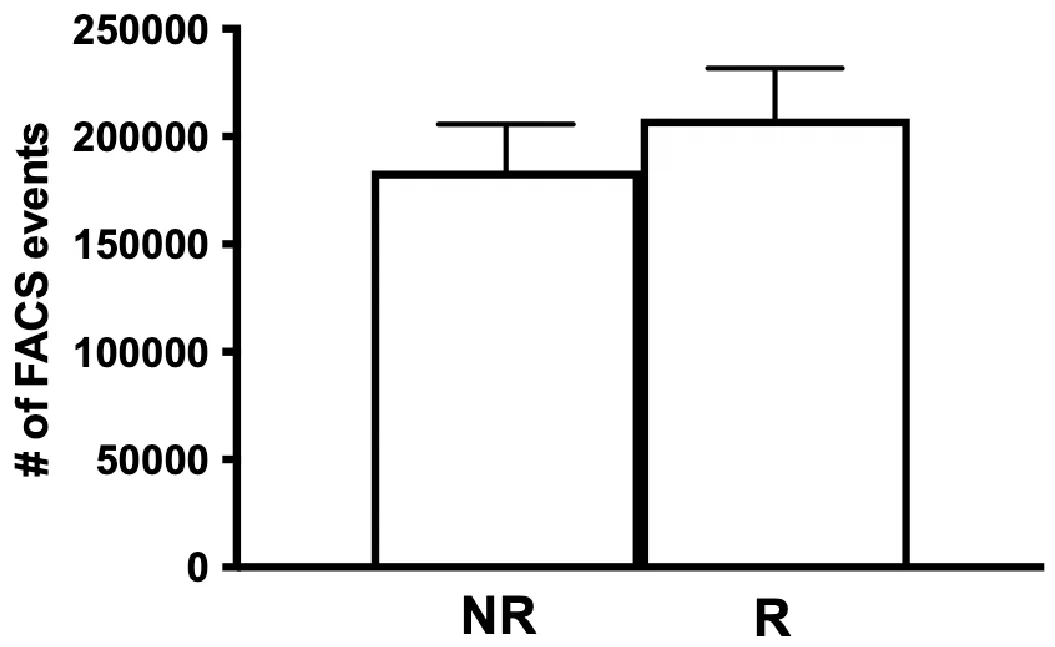
Plasma counts of extracellular plasma particles before the treatment with valproate (per μl). NR - non-responders, R-R-responders.

## Discussion

The salient findings of the current pilot study are: 1) Altered plasma levels of CRP but not altered counts of plasma particles predict response to VPA in patients with AUD/BD, 2) Treatment with VPA alters plasma levels of inflammatory cytokines but does not affect counts of plasma particles.

Treatment of AUD in a personalized manner is yet to be achieved. AUD rarely presents as an isolated disease and is commonly associated with other psychiatric illnesses, most often – with BD (12, 52). While efficacy of mood stabilizers in isolated AUD remains the topic of debate (18, 53), this class of medication may be particularly beneficial for patients with combined AUD and BD. We previously reported that patients with AUD/BD treated with VPA had a lower proportion of heavy drinking days and fewer drinks per heavy drinking day (17). We could not determine, however, why certain patients respond to VPA while others remain refractory. Difficulty in finding predictors for AUD/BD may result from the complexity of the intertwined pathogenetic mechanisms. AUD and BD are polygenic diseases which develop and progress as a result of numerous interactions between various genetic and environmental factors. Even in a non-comorbid AUD, there are currently no predictors of clinical response to such commonly used medications as naltrexone or acamprosate. Likewise, no algorithms have been developed to address which subpopulations of patients with BD would respond to a particular mood stabilizer or atypical antipsychotic.

In the current study, we found that pro-inflammatory cytokines may serve as a marker of clinical response to VPA. Specifically, we determined that responders to VPA have increased baseline plasma levels of CRP. We also observed that VPA decreases CRP and increases the metalloprotease MMP-10. Mechanisms by which VPA imparts an anti-inflammatory effect are not elucidated but are likely related to an ability of VPA to function as an inhibitor of histone deacetylases (54) thereby orchestrating the expression of multiple genes. These epigenetic properties of VPA recently again came under scrutiny, as clinical benefits were discerned in repurposing VPA for the treatment of systemic inflammation in patients with COVID-2019 (55, 56).

We also addressed if patients with AUD/BD have altered counts of plasma particles. It has been known that various classes of extracellular particles are closely linked to the progression of a pro-inflammatory state. Treatment of endothelial cells with CRP causes the release of extracellular vesicles (57). Likewise, production of extracellular particles is triggered by the staphylococcal superantigen-like protein 5 (58). Reversely, circulating particles induced expression of cyclooxygenase-2 and production of prostacyclins in endothelial cells (32). In our study, although responders to VPA had elevated levels of inflammatory indices, we did not observe the difference in counts of plasma particles between responders and non-responders suggesting that extracellular particles in plasma are unlikely to be reliable predictors of clinical response to VPA.

That inflammation is a marker of clinical response to VPA may be related to the involvement of inflammation in both AUD and BD. Increased levels of CRP were reported in men who consume excessive amounts of alcohol (59, 60). Furthermore, patients with AUD were demonstrated to have altered systemic concentrations of multiple cytokines (reviewed in (61)). Several studies established the association between BD and increased pro-inflammatory cytokines including CRP, IL-2, IL-6, and TNF (22–27). One may speculate that elevated levels of pro-inflammatory markers in AUD/BD are not mere bystanders but rather a pathogenetic link, and VPA is effective in these patients due to its anti-inflammatory action.

We suggest that the identified set of pro- and anti-inflammatory cytokines may represent only a part of a putative prognostic panel, and further search for other predictors of the response to VPA in patients with AUD/BD is warranted. RNA sequencing studies of brain from patients with BD showed that only a handful of genes were differentially expressed (62, 63). AUD, on the contrary, is accompanied by genome-wide changes in brain transcriptome (64–67); VPA also induces broad changes in gene expression (43, 68). Potential predictors may be represented by perturbed expression of non-coding RNAs, altered DNA methylation profiles, and chromatin remodeling (latter may be particularly attractive given that VPA is a histone deacetylase inhibitor). Development of a comprehensive prognostic panel will allow to find patients with co-occurring AUD/BD who will likely benefit from VPA.

Our pilot study has a few limitations. Sample size is relatively small, and more patients should be recruited for a replication cohort as well as for a search of novel prognosticators. Furthermore, data from our cohort may not be highly generalizable. Recent study demonstrated that Han Chinese population has a much lower prevalence of low-grade inflammation in BD (47), so that other ethnic groups may have to be studied separately.

## Data Availability

The original contributions presented in the study are included in the article/supplementary material. Further inquiries can be directed to the corresponding author.
